# Midfoot “H-Plate” for operative fixation of posterior wall acetabular fractures: a technical trick and case series

**DOI:** 10.1007/s00590-025-04579-9

**Published:** 2025-11-20

**Authors:** C. Julian Clark, Johnathan W. Riley, Alice McCraney, Isaac J. Spears, Drew P. Melancon, Tyler McGee, S. Austin Childress, Peter N. Mittwede, John Morellato, Patrick F. Bergin, Dillon C. O’Neill, Bradley A. Foulke, Matthew L. Graves, George V. Russell

**Affiliations:** 1 Department of Orthopaedic Surgery and Rehabilitation, University of Mississippi Medical Center, Jackson, USA; 2 Department of Orthopaedic Surgery, Indiana University Health, Indianapolis, USA; 3 Department of Orthopaedic Surgery, Mayo Clinic Health System, Eau Claire, USA; 4 Department of Orthopaedic Surgery, University of Pittsburgh Medical Center, Pittsburgh, USA

**Keywords:** Acetabulum, Posterior-wall fractures, Marginal impaction, Hip survivorship, Orthopaedic trauma

## Abstract

**Background:**

Acetabular fractures involving the posterior wall remain challenging to treat, with no gold standard fixation method established. We describe a novel technique using Synthes 2.7 mm non-locking plates originally designed for the midfoot.

**Methods:**

Retrospective review of 120 patients with posterior wall acetabular fractures treated with H-plates at a Level-1 trauma center (2012–2021). Patient demographics, operative parameters, and outcomes were analyzed.

**Results:**

The cohort was predominantly male (67.5%) with motor vehicle collisions as the primary mechanism (89.1%). Forty-seven patients (39.1%) received isolated H-plate fixation. Operative time was significantly shorter with isolated H-plates versus additional hardware (2.25 ± 0.60 vs 2.70 ± 0.91 h; *p* = 0.003). No patients required reoperation for fixation failure. Total hip arthroplasty was required in 9.2% of patients.

**Conclusion:**

The H-plate offers a simplified approach to posterior wall fixation with promising preliminary results, though longer follow-up is needed.

## Introduction

Posterior wall acetabular fractures represent one of the most common and challenging injury patterns in orthopedic trauma [1–6]. Despite decades of surgical refinement, these injuries continue to be associated with unsatisfactory clinical outcomes and high rates of post-traumatic arthritis [4, 7–12]. The critical importance of achieving anatomic or near-anatomic reduction has been consistently demonstrated across multiple studies, directly correlating with improved long-term hip survivorship [7, 12–14].

The standard fixation construct, popularized by Letournel and Judet, combines independent lag screw fixation with an anatomically contoured pelvic reconstruction plate that acts as a buttress against posterosuperior displacement of the posterior wall fragment [10]. However, this traditional approach presents several technical challenges. Manual contouring of reconstruction plates is time-intensive and requires significant surgical experience. Lag screw placement in peripheral or comminuted fracture patterns risks further fragmentation of already compromised bone. Additionally, the bulky nature of reconstruction plates often necessitates extensive soft tissue dissection, potentially increasing surgical morbidity.

Alternative fixation strategies have emerged to address these limitations. Some authors advocate for buttress plating alone without lag screws [15], while others propose “spring plates” to increase cortical contact in comminuted patterns [16–18]. Despite these innovations, all described techniques rely heavily on hand-contoured reconstruction plates as the primary fixation method.

We present the use of the Depuy Synthes 2.7 mm Hind/Midfoot plate (“H-plate”) for posterior wall acetabular fractures. This implant, originally designed for midfoot fractures, offers unique advantages including anatomic contouring that matches the posterior wall without modification, rounded edges that minimize soft tissue irritation, and robust construction that allows isolated use without supplemental fixation in many cases.

## Methods

Following Institutional Review Board (IRB) approval, we identified patients treated for posterior wall acetabular fractures using CPT code 27,226 from June 2012 to June 2021. Inclusion criteria were isolated posterior wall fractures treated with H-plates. Patients with concomitant ipsilateral hip girdle injuries were excluded to minimize operative time variability. Demographics, operative parameters, and outcomes were retrospectively collected from electronic medical records.

### Surgical technique

**Fig. 1:**
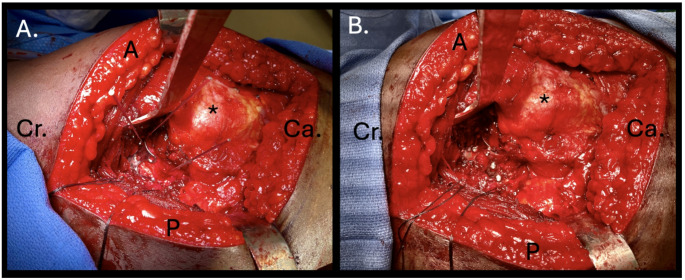
Intraoperative photographs. **A** The fracture is exposed through a standard Kocher-Langenbeck exposure to the posterior acetabular surface. Deep dissection can be limited both cranially and caudally when using the H-plate as an isolated implant. The fracture is wired into a reduce position and the H plate is wired in place without pre-bending prior to screw placement. **B** Final construct fixation demonstrating screw placement in a dual H-plate construct. *− Greater trochanter, Cr. – Cranial, Ca. – Caudal, A. – Anterior, P.– Posterior

**Fig. 2:**
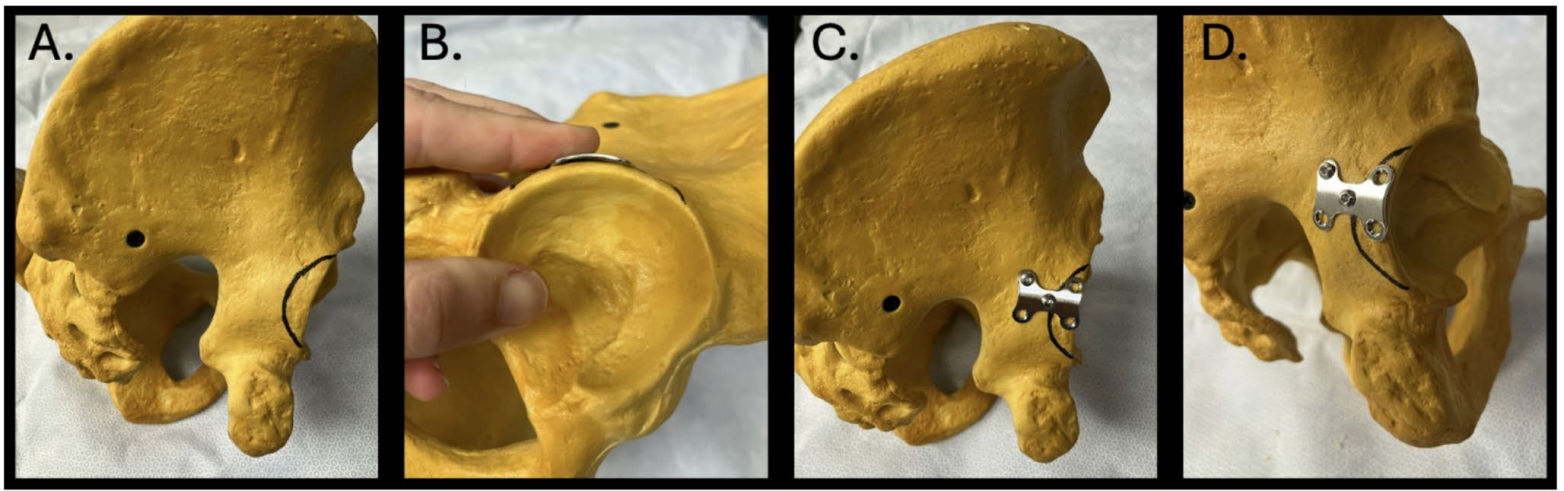
Implant position. **A** Sawbones model demonstrating a peripheral posterior wall variant fracture line **B** The design of the H-plate matches the radius of curvature of the posterior wall. It can be placed easily without pre-contouring. **C** and **D** Final construct placement demonstrating standard screw configuration and plate position. The plate is optimally positioned immediately at the acetabular bone edge with the goal of buttressing against posterosuperior translation of the fracture fragment. The blunted edges of the plate minimize irritation of the acetabular labrum and other soft tissues

Patients with posterior wall acetabular fractures underwent a Kocher-Langenbeck exposure in the lateral position. Compared to a standard Kocher-Langenbeck exposure, minimal dissection of the gluteus minimus and ischial tuberosity was performed when using H-plates in isolation. The fracture was identified and cleaned with care to expose only the fracture edges required for establishing an anatomic cortical read where possible. The fracture was then typically reduced with two ball spike pushers and fastened with Kirschner wires (K-wires) in a reduced position. The H-plate was then inserted, positioned, and secured in place with two K-wires to prevent rotation during screw insertion. (Fig. 1) The plate inherently adheres well to the posterior wall without modification and is rarely manually contoured prior to insertion. The plate was typically positioned such that the lateral two holes of the plate abut the acetabular rim. (Fig. 2) Positioning along the acetabular rim was confirmed with a free K-wire to ensure that no metal protruded lateral to the acetabular labrum. The H-plate was then fixed to the bone through the central hole followed by one or both medial holes depending on the patient’s bone quality. Though the plate was designed for 2.7 mm screws, 3.5 mm cortical screws are typically used. No lag screw fixation was used given the stout buttress created by the H-plate. Depending on the size of the fracture, multiple H-plates were frequently used. Additional fixation, most commonly a 3.5 mm contoured pelvic reconstruction plate placed over the H-plate(s), was used based on fracture characteristics and the discretion of the treating surgeon.

Postoperatively, patients were instructed to be toe-touch weightbearing on their affected lower extremity for 6–12 weeks. Physical therapy was not routinely utilized except for patients requiring aid with basic ambulation in the postoperative period. Patients received radiation therapy for heterotopic ossification prophylaxis when possible. For patients who were not candidates for or who declined radiation prophylaxis, indomethacin was prescribed for heterotopic ossification prophylaxis. Post-operative checks occurred at 2, 6, 12 weeks, 6, and 12 months for patients who presented for scheduled follow-up.

## Results

Of 314 identified patients, 160 (50.9%) were treated with H-plates. After excluding 40 patients with ipsilateral injuries, 120 patients comprised our final cohort. The population was predominantly male (81/120, 67.5%) with motor vehicle collisions as the primary mechanism (107/120, 89.1%). Marginal impaction was present in 26 cases (21.7%). (Table 1).

**Table 1:** Baseline demographic data

Characteristic		Value
Age at time of surgery in years, mean (SD)		33.9 (14.3)
*Gender, n (%)*		
	Male	81 (67.5)
	Female	39 (32.5)
BMI, mean (SD)		30.9 (7.7)
*Comorbidities, n (%)*		
	Tobacco use	45 (37.5)
	Alcohol use	41 (34.1)
	Recreational drug use	21 (17.5)
	Immunocompromised status	1 (0.8)
	Hypertension	27 (22.5)
	Diabetes mellitus	5 (4.2)
	COPD	3 (2.5)
	ESRD	2 (1.7)
	Vascular disease	
	CAD	2 (1.7)
	TIA/stroke	1 (0.8)
*Mechanism of injury, n (%)*		
	MVC/MCC	107 (89.1)
	Fall less than 10 feet	7 (5.8)
	Fall greater than 10 feet	1 (0.8)
	Other	5 (4.1)

**Fig. 3:**
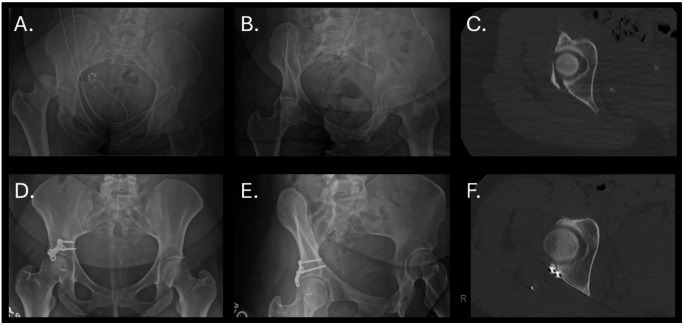
Case example. **A** Anteroposterior radiograph of an isolated posterior wall fracture dislocation. **B** Obturator oblique radiograph of the posterior wall fracture following closed reduction. **C** Axial computed tomography demonstrating cranial and peripheral nature of posterior wall fracture. **D** Post-operative anteroposterior radiograph. **E** Post-operative obturator oblique radiograph. **F** Post-operative axial computed tomography demonstrating near anatomic wall reduction and peripheral plate positioning

Forty-seven patients (39.1%) received isolated H-plate fixation, while 73 (60.8%) had additional hardware. (Fig. 3) Median follow-up was 4.0 months (range 0–102.3 months). Isolated H-plate use was associated with significantly shorter operative times (2.25 ± 0.60 vs 2.70 ± 0.91 h; p = 0.003) and reduced blood loss (263.0 ± 93.3 vs 325.1 ± 182.2 cc; p = 0.033).

No patients required reoperation for fixation failure. Five patients (4.2%) required reoperation for infection (4 cases) or hardware removal (1 case). Total hip arthroplasty was performed in 11 patients (9.2%), including only 2 patients (4.3%) from the isolated H-plate group.

## Discussion

The H-plate technique addresses several limitations of traditional posterior wall fixation. Its pre-contoured design eliminates time-consuming manual shaping while providing optimal anatomic fit. The rounded edges and low profile minimize soft tissue irritation and allow placement directly at the acetabular rim without pre-operative modification. The robust construction creates effective buttress support, often eliminating the need for supplemental lag screws that risk fragment comminution in peripheral fracture patterns.

For comminuted injuries, the H-plate’s broad footprint can capture multiple cortical fragments with a single implant, significantly simplifying fixation of complex patterns. The simplified technique also reduces required soft tissue dissection, potentially decreasing operative morbidity and infection risk.

Our results demonstrate feasibility of this approach with outcomes comparable to published literature despite limited follow-up [11, 19]. The 39.1% of patients treated with isolated H-plates experienced shorter operative times and reduced blood loss, suggesting potential advantages of the simplified technique. However, selection bias likely influences these results, as surgeons may preferentially use isolated fixation for less complex fractures.

### Limitations

This study has several important limitations. The retrospective design and lack of control group limit our ability to demonstrate superiority over traditional methods. Short median follow-up (4 months) is inadequate for assessing long-term hip survivorship, which typically requires years to evaluate meaningfully. Treatment selection was surgeon-dependent rather than protocol-driven, introducing potential confounding between groups based on fracture complexity. The wide follow-up range reflects challenges in maintaining long-term patient contact in our catchment area.

Additionally, we lack functional outcome measures, detailed radiographic analysis of reduction quality, and comprehensive complication assessment beyond major reoperations. These limitations prevent definitive conclusions about the technique’s efficacy compared to established methods.

## Conclusion

The H-plate represents a novel approach to posterior wall acetabular fracture fixation that may offer advantages over traditional reconstruction plating, including simplified technique, reduced operative time, and elimination of manual contouring requirements. The implant’s design characteristics make it particularly suitable for peripheral and comminuted fracture patterns that are challenging to treat with conventional methods.

While our preliminary results are encouraging, this study should be interpreted as proof of concept rather than definitive evidence of superiority. The technique appears safe and feasible, with complication rates comparable to published literature. However, prospective studies with appropriate control groups, longer follow-up, and comprehensive outcome measures are essential to validate these findings.

Practicing trauma surgeons may benefit from familiarity with this technique, particularly for challenging posterior wall fracture patterns where traditional fixation methods may be suboptimal. The H-plate’s ready availability in most trauma centers makes it an accessible option for surgeons seeking alternatives to complex reconstruction plating techniques.

Future research should focus on prospective comparative studies with standardized outcome measures, long-term follow-up assessment of hip survivorship, and detailed analysis of which fracture patterns are most suitable for this fixation method. Only through such rigorous investigation can the true value of this technique be established.

## Data Availability

No datasets were generated or analysed during the current study.
